# Analysis of Correlation Between the Core Competency Status and Spiritual Health of Nursing Intern Students

**DOI:** 10.1155/jonm/3522503

**Published:** 2026-02-02

**Authors:** Limei Zhang, Xiaoying Zhong, Jing Liu, Tingting Yuan, Wenli Yang, Meng Wang, Ping Yuan, Xing Ding

**Affiliations:** ^1^ School of Nursing, Chengdu Medical College, Chengdu, Sichuan, 610500, China, cmc.edu.cn; ^2^ Sichuan Provincial Key Laboratory of Philosophy and Social Sciences for Intelligent Medical Care and Elderly Health Management, Chengdu, Sichuan, 610500, China; ^3^ Sichuan Provincial Center for Mental Health, Sichuan Provincial People’s Hospital, School of Medicine, University of Electronic Science and Technology of China, Chengdu, Sichuan, 610031, China, uestc.edu.cn; ^4^ Anesthesia and Pain Department, Chinese People’s Liberation Army Western Theater Command General Hospital, Chengdu, Sichuan, 610083, China

**Keywords:** health, nursing, professional competence, spirituality, students

## Abstract

**Aims:**

This study aimed to investigate the current status of nursing intern students’ core competencies and their relationship with spiritual health.

**Background:**

Cultivating the core competencies of nursing intern students is a significant educational objective. As nursing intern students transition into clinical practice, they encounter heightened levels of stress, which can adversely impact both physical and mental health, potentially hindering the development of their competencies. In recent years, there has been an increasing interest in spiritual health, which can help individuals cope with stress, promoting positive physical and mental health outcomes. However, the relationship between spiritual health and the core competencies of nursing intern students remains unclear.

**Methods:**

Convenience sampling was used to recruit 312 nursing intern students from five tertiary hospitals in China. Data regarding sociodemographic characteristics, spiritual health, and core competencies were collected using online questionnaires from September 2022 to January 2023.

**Results:**

The overall average score of core competencies of nursing intern students (149.70 ± 19.09) was at a medium level, among which the score of critical thinking and reasoning ability was the lowest (11.28 ± 2.02). Univariate analysis shows that factors such as place of birth, reasons for choosing the nursing major, and employment intentions have an impact on the core competencies of nursing intern students. Correlation analysis indicated that the core competencies of nursing intern students were positively correlated with their spiritual health (*r* = 0.687, *p* < 0.01). Hierarchical multiple regression analysis showed that spiritual health explained 42.9% of the total variance in core competencies (△*R* ^2^ = 0.429, *p* < 0.01).

**Conclusions:**

This study shows that the core abilities of nursing intern students need to be improved, especially in terms of critical thinking and reasoning skills. In addition, their spiritual health level will have an impact on the development of core competencies.

**Implications for Nursing Management:**

The results of this study suggest that nursing managers and educators should attach importance to the spiritual health level of nursing intern students, take timely intervention measures, and enhance the core competencies of nursing intern students. This is the key to promoting the professional development of clinical nursing and facilitating the high‐quality development of nursing.

## 1. Introduction

As healthcare delivery becomes increasingly complex, with a heightened emphasis on effective and patient‐safe nursing practice, the demand for nursing core competencies has increased [1]. Core competencies encompass the knowledge, skills, judgment, and personal attributes that nurses must possess to provide safe and ethical nursing care [2]. These competencies are regarded as fundamental for evaluating nursing proficiency [3] and represent the comprehensive skill set required to perform clinical tasks effectively [4]. Nursing intern students are the reserve force of future nurses; therefore, the cultivation of core competencies in nursing intern students has become an essential nursing education objective [5].

Clinical practice is an integral part of nursing education [6] and can help nursing intern students apply nursing knowledge to clinical nursing work, effectively improving general competencies. Hu et al. found through a longitudinal study that nursing intern students’ competencies need to be developed during clinical practice [7]. Hence, it is imperative for nursing intern students to cultivate core competencies across various domains such as clinical biomedical science, general clinical skills, critical thinking and reasoning, ethics and accountability, caring practices, and lifelong learning throughout their clinical experiences [8, 9]. Studies have shown that the average core competencies level of nursing intern students is at an intermediate level and requires further improvement [10, 11]. Inadequate nursing core competencies not only affect patient safety [12] but may also diminish nursing intern students’ satisfaction with their clinical placements and expectations regarding their learning environments. This can adversely impact their willingness to pursue careers in nursing postgraduation [6, 13]. This makes it important to focus on the weak core competencies of nursing intern students and to determine how to improve them.

Previous research found that practice stress was negatively associated with nursing competencies [14]. In contrast, factors such as psychological resilience [5], clinical adaptability [5], professional identity [10], engagement in studying [10], and the ability to learn autonomously [11] have been shown to have positive impacts on core competencies. Caring is widely regarded as the essence of nursing [15], and Watson’s caring theory’s “caring factors” and “caring process” indicate that care for the self and for the one being cared for determines the competencies of professional nurses [16]. Evidence also suggests that self‐care can help nursing intern students manage and cope with stress, develop adaptive capacity, and enhance professional identity [17], all of which can improve self‐directed learning, learning engagement, and self‐efficacy, thereby contributing to the development of core competencies [10, 11, 18, 19]. Self‐care refers to life activities aimed at optimizing one’s health, improving both health and personal growth [20, 21]. These activities include taking responsibility for one’s health, using stress management techniques, and engaging in spiritual growth practices [22, 23]. From the perspective of Watson’s theory, the development of caregiving ability should be based on the nurse’s internal resources, with self‐care being the core approach to building these resources [24].

Spirituality is an important component of well‐being [25]. According to Watson’s theory, spiritual health is defined as an individual’s intrinsic energy that fosters caring practices, cultivated through a harmonious connection with both the internal and external environment and a sense of meaning in life [26]. Specifically, spiritual health is manifested as an individual’s affirmation of the meaning and value of their own life, a harmonious connection with others, society, and nature, and the possession of inner energy, as well as a state of being able to transcend limitations [27]. As one of the key aspects of health [28], spiritual health is critical to the self‐care of an individual. Spiritual health has been shown to promote spiritual growth, defined as the development of inner resources for the cultivation of personal values and connection with a higher power [23]. Additionally, spiritual health is the newest dimension of harmonizing physical, psychological, and social aspects of health [29]. Recent studies have demonstrated that spiritual health can assist individuals in managing clinical stress, reducing depression, and promoting a healthier overall lifestyle [30, 31].

Nursing intern students encounter significant stress when transitioning to clinical studies [32]. Stressors include the gap between practice and theory [33], unfamiliar clinical environments, patient death [34], and harm to patients or themselves [32]. According to Watson’s theory, when the spiritual health of nursing intern students is compromised due to stress, their ability to provide care may be replaced by “dehumanized” mechanical operations [35]. This state is easy to cause chronic health conditions and work burnout for nursing staff, which is found to have a negative impact on the development of competence [36]. Optimal health in nursing staff is vital for the provision of nursing care [28]. Chiang also noted that the spiritual health of nursing intern students should be emphasized in the education process to better help them cope with the challenges of the clinical setting [30].

In 2023, the National Health Commission proposed in the “*Action Plan for Further Improving the Level of Nursing* Services (2023–2025)” that medical institutions should take strengthening the “Three Basics and Three Strictness” as the entry point, consolidate the clinical skills of nurses, promote the development of clinical nursing specialties and the cultivation of nursing talents, and contribute to the high‐quality development of the nursing cause [37]. In recent years, the iceberg model of competence has been widely applied in the research of nursing education management and the construction of clinical ability index systems for nursing staff and has achieved satisfactory results [38, 39]. According to this model, nursing ability not only encompasses knowledge and skills as tangible components, that is, the “visible part of the iceberg,” but also includes potential personal traits such as motivation, traits, and self‐image, that is, the “underwater part of the iceberg” [40], which is the core of ability [38]. Meanwhile, the model points out that motivation and traits are the internal driving forces of an individual’s external behavior, while self‐image is formed based on the sense of identity generated by values. These inner traits of an individual are closely related to their spiritual state and spiritual health level [41]. They promote the development of inner traits, and inner traits in turn influence professional abilities. Considering the significance of core competencies in nursing and the role of one’s inner spirituality in this regard, this study hypothesizes that the spiritual health of nursing intern students is an important predictor of core competencies. Therefore, this study aims to explore the correlation between spiritual health and the core competencies of Chinese nursing intern students.

## 2. Methods

### 2.1. Design and Participants

A descriptive, cross‐sectional design was used. In this study, 312 nursing intern students were recruited through convenience sampling from five tertiary hospitals in Sichuan Province, China, from September 2022 to January 2023. Inclusion criteria were nursing intern students who were (1) at least 18 years old, (2) full‐time nursing intern students who had not graduated, (3) in clinical practice lasting longer than 3 months, and (4) volunteered to participate in the study. Students who were absent from their posts during the investigation period were excluded.

The sample size in this study was obtained using the rough estimation approach [42], in which the sample size is 10 times the number of independent variables. With 19 independent variables in this study, the sample size was calculated to be at least 209 instances, assuming that 10% of questionnaires would be invalid.

### 2.2. Instruments

#### 2.2.1. Demographic Questionnaire

The demographic questionnaire was self‐made by the research team and included factors such as gender, age, education level, whether they were from an only child family, birthplace, reason for majoring in nursing, employment intention, and the length of clinical practice.

#### 2.2.2. Spiritual Health Scale‐Short Form (SHS‐SF)

The SHS‐SF, developed by Chinese scholar Hsiao et al., was used to measure the spiritual health of nursing intern students [43]. The SHS‐SF consists of 24 items scored on a 5‐point Likert scale (1 = *totally disagree*, 5 = *totally agree*). This scale is divided into five subscales: connection with others (four items), meaning from life (six items), transcendence (six items), religious attachment (four items), and self‐understanding (four items). The total score ranges from 24 to 120; the higher the score, the better the spiritual health condition. The overall Cronbach’s α coefficient of the scale was 0.93, and Cronbach’s α coefficients of the subscales ranged from 0.88 to 0.90. Confirmatory factor analysis showed that each fitting index was good (*χ* ^2^/df = 2.80, GFI = 0.95, RMSEA = 0.059). The test–retest reliability was 0.77, and the overall content validity index (CVI) ranged from 0.83 to 1.0, confirming that the scale had excellent psychometric properties [44]. This scale is widely applied. Therefore, this study adopted this scale to assess the spiritual health status of Chinese nursing intern students. In this study, Cronbach’s *α* coefficients varied from 0.85 to 0.93.

#### 2.2.3. Chinese Version of the Competency Inventory for Nursing Students (CINS)

The CINS, developed by Hsu and Hsieh and translated by Liao et al., was used to assess the core competencies of nursing intern students [8, 45]. This scale consists of 38 items and has 6 dimensions, including clinical biomedical science (5 items), general clinical skills (6 items), critical thinking and reasoning (3 items), caring (5 items), ethics and accountability (14 items), and lifelong learning (5 items). The Likert 5‐level scoring method is adopted, the total score ranges from 38 to 190, and the higher the score, the stronger the core ability of the nursing intern students. The overall Cronbach’s α of this scale was 0.96, Cronbach’s α of each dimension ranged from 0.82 to 0.95, the Guttman half‐reliability was 0.83, and the test–retest reliability was 0.74, demonstrating good internal consistency and stability, which showed excellent psychometric properties [45]. In this study, Cronbach’s α varied from 0.82 to 0.97.

### 2.3. Data Collection

Following ethical approval, data collection was conducted. After receiving consent from the head of the surveyed hospital, one or two investigators were assigned to each hospital. These investigators sent notification via a social media application (i.e., WeChat) informing the participants of the study purpose and the time required for survey completion (10–20 min), as well as the inclusion and exclusion criteria. This information was also included in the instructions of the questionnaire. After obtaining participant consent, all electronic questionnaires were collected through an electronic data collection tool called Wen Juan Xing. Participants’ identity information was not collected, and their privacy was strictly maintained. All questions were mandatory, and incomplete submissions were systematically blocked by the platform. Following data collection, two researchers independently entered and cross‐verified the responses to ensure accuracy. The following exclusion criteria were rigorously applied: the questionnaire filled in less than 3 min, the questionnaire with obviously inconsistent personal information of the respondents (e.g., age that is obviously too young or old), and the questionnaire with contradictory answers.

### 2.4. Ethical Considerations

This study was approved by the Ethics Committee of Chengdu Medical College, China (2021, No. 09). All participants were provided with information about the study, including the study’s purpose. Participants were asked to complete an online questionnaire independently and anonymously.

### 2.5. Data Analysis

All data analyses were performed using the Statistical Package for the Social Sciences (SPSS; Version 26.0). Quantile–quantile plotting was used to examine normal distributions for spiritual health and core competencies. Descriptive analyses were performed on all data (percentages, means, and standard deviations [SDs]). Differences in core competencies were analyzed using a *t*‐test and one‐way analysis of variance (ANOVA). To judge the actual importance of the difference, the effect size between two groups was expressed as the *d*‐value (Cohen’s d), while the effect size between multiple groups was expressed as *η* ^2^ (eta‐squared). A 95% confidence interval was also provided. For multiple comparisons with statistical significance in one‐way ANOVA, the LSD‐t test was used with Bonferroni correction for multiplicity. Correlations between spiritual health and core competencies were tested using Pearson’s correlation analysis. Finally, a hierarchical multiple regression analysis was performed to identify the factors significantly associated with core competencies. Before conducting the regression analysis, the Durbin–Watson (D–W) test was used to check for residual autocorrelation by testing the independence of the residuals. Next, homogeneity of variance was tested using a residual scatter plot. Additionally, normality of the data was assessed using a P–P plot and a residual histogram. Finally, multicollinearity among the independent variables was checked using the variance inflation factor and tolerance to ensure methodological appropriateness. *p* < 0.05 was considered statistically significant.

## 3. Results

### 3.1. Participant Descriptions

This study recruited 328 nursing intern students, 312 of whom completed the survey (a response rate of 95.12%). Table 1 presents the participants’ demographic variables. 91.3% were female, and most were aged between 21 and 24 years. Additionally, 71.2% were undergraduate students, 66.3% had siblings, and 48.7% were from rural areas. Over one‐third of the nursing intern students voluntarily chose a nursing career, nearly half expressed an interest in clinical nursing, and 59.3% had internships lasting between 3 and 6 months. Additionally, univariate analysis shows statistically significant differences in core competencies among nursing student interns based on their place of birth, reasons for choosing nursing as a major, and employment intentions. All differences were close to moderate effect size (*p*  < 0.05; *η* ^2^: 0.01∼0.06, Table 1).

**TABLE 1:** Comparison of core competencies among nursing intern students with different sociodemographic characteristics (*n* = 312).

Variables	*n*	%	Core competencies				Effect size		
			Mean ± SD	*t*/*F*	*p*	LSD	*d*/*η*2	95% CI	
								Lower	Upper
Sex				−0.346	0.729		0.503	−0.465	0.325
Male	27	8.7	148.48 ± 22.87						
Female	285	91.3	149.81 ± 18.74						
Age (years)				0.369	0.692		0.002	< 0.001	0.019
18–20	69	22.1	149.57 ± 23.67						
21–24	240	76.9	149.85 ± 17.67						
≥ 25	3	1.0	140.33 ± 13.01						
Educational level				0.366	0.778		0.004	< 0.001	0.017
Technical secondary school	20	6.4	150.85 ± 20.29						
Associate	65	20.8	147.51 ± 21.44						
Bachelor	222	71.2	150.23 ± 18.50						
Master	5	1.6	149.80 ± 4.97						
One child				0.436	0.663		0.503	−0.183	0.287
Yes	105	33.7	150.36 ± 16.27						
No	207	66.3	149.36 ± 20.41						
Place of birth				4.416	0.013		0.038	0.001	0.069
Urban (A)	64	20.5	155.95 ± 19.41		0.033^AB^	A > B			
Town (B)	96	30.8	148.16 ± 14.67		0.964^BC^	A > C			
Rural (C)	152	48.7	148.04 ± 20.91		0.016^AC^				
Reason for majoring in nursing				3.288	0.012		0.041	0.002	0.082
Personal preference (A)	106	34.0	155.00 ± 16.46		0.031^AB^	A > B			
Parental options (B)	76	24.4	147.68 ± 20.40		0.918^BC^	A > C			
Higher education transfer (C)	51	16.3	147.33 ± 17.69		0.049^AC^	A > D			
Employment implications (D)	43	13.8	146.65 ± 16.07		0.045^AD^	A > E			
Other reasons (E)	36	11.5	145.33 ± 25.41		0.024^AE^				
Employment intention				4.061	0.003		0.05	0.007	0.094
Clinical nursing (A)	154	49.4	151.29 ± 17.32		0.003^AE^	E < A			
Pharmaceutical company (B)	21	6.7	155.14 ± 8.84		0.019^BE^	E < B			
Further education (C)	36	11.5	152.58 ± 22.12		0.022^CE^	E < C			
Nursing school (D)	60	19.2	149.02 ± 16.40		0.117^DE^				
Other jobs (E)	41	13.1	139.39 ± 26.09						
Length of clinical practice				0.144	0.866		0.001	< 0.001	0.011
3–6 months	185	59.3	149.99 ± 17.05						
7–10 months	96	30.8	149.68 ± 23.51						
> 10 months	31	9.9	148.00 ± 15.58						

Note: The superscript symbols in the table represent general information codes. For example, in “Place of birth,” “Urban” is represented by “A,” “Town” is represented by “B,” and “Rural” is represented by “C.” “0.033AB, and A > B” indicates that in the LSD comparison, the core competencies of nursing intern students born in cities are higher than that of nursing intern students born in towns. Other statistically significant general information from univariate analysis is also represented in the same way and compared using LSD.

Abbreviation: SD, standard deviation.

### 3.2. Spiritual Health and Core Competencies

The quantile–quantile plot shows that the data for spiritual health and core competencies are roughly normally distributed. Therefore, mean values and SDs are used to describe the status of these two variables. As shown in Table 2, the overall average score for spiritual health is 91.33 ± 13.91. Among these, the subscale score for social interaction with others is the highest, while the subscale score for religious attachment is the lowest. The overall average score for core competencies is 149.70 ± 19.09. The highest scores are for ethics and responsibility, and the lowest scores are for critical thinking and reasoning.

**TABLE 2:** Spiritual health and core competencies among nursing intern students (*n* = 312).

Variables	Domain	Min	Max	Mean ± SD (item mean score)
Spiritual health	Connection to others	4.00	20.00	16.23 ± 3.14 (4.06 ± 0.79)
	Meaning derived from living	6.00	30.00	24.14 ± 4.08 (4.02 ± 0.68)
	Transcendence	6.00	30.00	23.34 ± 4.33 (3.89 ± 0.72)
	Religious attachment	4.00	20.00	11.51 ± 4.53 (2.88 ± 1.13)
	Self‐understanding	4.00	20.00	16.12 ± 2.74 (4.03 ± 0.69)
	Overall	24.00	120.00	91.33 ± 13.91 (3.81 ± 0.58)

Core competencies	Clinical biomedical science	5.00	25.00	18.90 ± 3.07 (3.78 ± 0.61)
	General clinical skills	6.00	30.00	22.99 ± 3.50 (3.83 ± 0.58)
	Critical thinking and reasoning	3.00	15.00	11.28 ± 2.02 (3.76 ± 0.67)
	Caring	5.00	25.00	19.61 ± 2.88 (3.92 ± 0.58)
	Ethics and accountability	14.00	70.00	56.90 ± 7.38 (4.06 ± 0.53)
	Lifelong learning	5.00	25.00	20.02 ± 2.93 (4.00 ± 0.59)
	Overall	38.00	190.00	149.70 ± 19.09 (3.94 ± 0.50)

*Note:* Min, minimum; Max, maximum.

Abbreviation: SD, standard deviation.

### 3.3. Relationship Between Spiritual Health and Core Competencies

As shown in Table 3, spiritual health was significantly correlated with core competencies (*r* = 0.687, *p* < 0.01). All of the subdomains of spiritual health had a significantly positive correlation with core competencies.

**TABLE 3:** Correlations between spiritual health and core competencies (*r* value, *n* = 312).

Variables	Domain	Core competencies
Spiritual health	Connection to others	0.562^∗∗^
	Meaning derived from living	0.678^∗∗^
	Transcendence	0.607^∗∗^
	Religious attachment	0.146^∗∗^
	Self‐understanding	0.635^∗∗^
	Overall	0.687^∗∗^

^∗∗^ *p* < 0.01.

### 3.4. Significantly Associated Factors of Core Competencies

Hierarchical regression analysis was used to explore the relationship between spiritual health level and the core competencies of nursing intern students. The D–W statistic was 2.013, indicating independent data. The residual scatter plot shows points distributed symmetrically around zero, suggesting that the homoscedasticity assumption is met. The P–P plot of residuals and the residual histogram show consistency with the normal distribution. The VIF value is below 5, and the tolerance value exceeds 0.2, indicating that there are no severe multicollinearity issues among the variables in the regression model. In Model 1, we used the core competency scores of nursing intern students as the dependent variable and input participants with statistically significant differences in place of birth, majoring in nursing, and employment intentions for the hierarchical regression analysis. After controlling for general characteristics, spiritual health was introduced as an independent variable into Model 2. Regression model results show that employment intentions, clinical nursing, pharmaceutical companies, continuing education, nursing schools, and spiritual health have a positive and significant impact on the core competencies of nursing intern students. Additionally, the hierarchical regression analysis indicates that spiritual health level is the strongest predictor of core competencies, explaining 42.9% of the total variance in core competencies (see Table 4).

**TABLE 4:** Hierarchical multiple regression analysis of core competencies (*n* = 312).

	Model 1						Model 2					
	*B*	SE	*β*	*t* (*p*)	Collinear statistics		*B*	SE	*β*	*t* (*p*)	Collinear statistics	
					Tolerance	VIF					Tolerance	VIF
Constant	139.253	3.803		36.615 (< 0.001)	0.834	1.199	57.273	5.688		10.069 (< 0.001)	0.814	1.228
D. Urban	6.872	2.831	0.146	2.427 (0.016)	0.815	1.227	1.576	2.079	0.033	0.758 (0.449)	0.813	1.23
D. Town	−1.469	2.505	−0.036	−0.586 (0.558)	0.335	2.983	−3.127	1.821	−0.076	−1.718 (0.087)	0.324	3.084
D. Personal preference	5.524	3.807	0.137	1.451 (0.148)	0.394	2.535	−2.884	2.809	−0.072	−1.027 (0.305)	0.393	2.547
D. Parental options	−1.757	3.873	−0.040	−0.454 (0.650)	0.476	2.102	−5.014	2.817	−0.113	−1.780 (0.076)	0.472	2.12
D. Higher education transfer	−0.395	4.093	−0.008	0.096 (0.923)	0.519	1.925	−4.942	2.983	−0.096	−1.657 (0.099)	0.519	1.928
D. Employment implications	−0.702	4.202	−0.013	−0.167 (0.867)	0.368	2.716	1.244	3.052	0.022	0.408 (0.684)	0.367	2.721
D. Clinical nursing	9.003	3.441	0.236	2.616 (0.009)	0.642	1.557	10.862	2.500	0.285	4.345 (< 0.001)	0.642	1.557
D. Pharmaceutical company	13.457	5.200	0.177	2.588 (0.010)	0.55	1.818	13.112	3.774	0.172	3.475 (0.001)	0.55	1.818
D. Further education	10.753	4.406	0.180	2.441 (0.015)	0.483	2.071	11.602	3.197	0.194	3.629 (< 0.001)	0.483	2.072
D. Nursing school	8.376	3.813	0.173	2.197 (0.029)			7.797	2.767	0.161	2.818 (0.005)	0.895	1.118
Spiritual health							0.951	0.058	0.693	16.483 (< 0.001)		
	*R* ^2^ = 0.097, adjusted *R* ^2^ = 0.067, △*R* ^2^ = 0.097 *F* = 3.236 (*p* = 0.001), D–W = 2.013						*R* ^2^ = 0.526, adjusted *R* ^2^ = 0.509, △*R* ^2^ = 0.429 *F* = 30.283 (*p* < 0.001), D–W = 2.145					

*Note:* D: dummy variables reference group: birthplace (rural), reason for majoring in nursing (other reasons), employment intention (other jobs).

## 4. Discussion

The core competencies of nursing intern students are directly related to the quality of nursing education and the core competencies of future nurses [46]. The results of our study indicated that spiritual health influenced the development of core competencies among nursing intern students. The findings of this study may contribute to the development of more targeted education and training to help nursing intern students improve their core competencies.

### 4.1. The Level of Core Competencies of Nursing Intern Students

The results indicated an intermediate level of core competencies among the current sample, consistent with previous studies of Chinese nursing intern students [10, 11]. The lowest score was obtained for the critical thinking and reasoning dimension among the six domains, which is consistent with previous literature studies [11, 47]. This may be due to barriers in translating knowledge into clinical practice. Nursing intern students may possess theoretical knowledge yet struggle to apply critical analysis and decision‐making in real‐world or complex clinical scenarios [48]. Critical thinking is essential for nurses to effectively manage complex health conditions and patient problems [49]. Therefore, it is important to improve critical thinking skills during the educational phase. Reflective practice is closely linked to critical thinking [50]. Research has demonstrated that cultivating critical thinking can be achieved through methods such as clinical mentors sharing their experiences [51, 52] and reflective journaling [49]. Additionally, because the development of critical thinking is highly dependent on the clinical context, contextualized teaching methods have unique advantages [53]. For example, simulation‐based learning (SBL) combines theoretical knowledge with practical operations and incorporates elements such as repeated practice, immediate feedback, assessment, and reflection to develop students’ thinking skills [54]. Therefore, based on the above evidence, nursing educators and clinical managers can adopt an integrated strategy. In clinical teaching, they can combine mentor experience sharing [51, 52] with reflective diary writing [49] to strengthen reflective practice. Simultaneously, they can use SBL simulation scenarios, employing immediate feedback and process debriefing to deepen thinking training [54–56]. This multidimensional, contextualized training model is expected to enhance nursing intern students’ critical thinking abilities more effectively.

### 4.2. Factors Influencing the Core Competencies of Nursing Intern Students

The ANOVA showed statistically significant differences in the core competencies of nursing intern students in terms of birthplace, reason for majoring in nursing, and employment intention. The core competencies of nursing intern students born in cities are significantly higher than those of nursing intern students born in rural areas, which is consistent with previous studies [57]. The differences in the core competencies of urban and rural nursing interns may be caused by multiple factors. First, urban areas have abundant medical resources and higher levels of medical and health services, impacting nursing intern students’ cognitive understanding of nursing [58]. Second, differences in early education resource accessibility mean urban nursing intern students have richer learning resources than their rural counterparts, such as more prevalent STEAM education and critical thinking training in urban schools [59, 60]. Therefore, nursing interns who receive an urban education may be better developed in creativity and critical thinking than those from towns and rural areas. These factors may contribute to the differences in core nursing competencies between urban and rural nursing intern students. Nursing intern students whose reason for majoring in nursing was “personal will” scored significantly higher in core competencies. This result is similar to those reported in an earlier study [47]. One possible reason for this is that students who voluntarily choose nursing may have a stronger professional identity or be more interested in the field. This may lead them to be more proactive in acquiring nursing knowledge and skills, thus improving their competencies [61]. However, it should also be noted that in the Chinese cultural context, family pressure is often intertwined with career choices [62]. In this study, nursing intern students who chose nursing as a major based on “personal willingness” may have been influenced by family factors, such as financial pressure. This confirms the complexity of motivation. In the future, it will be necessary to design a motivation‐type scale to distinguish the differences in the impact of motivations such as “passive choice” and “active acceptance” on core competencies. Taking this into account, nursing educators and clinical managers should emphasize the intrinsic interest and motivation of nursing intern students. They should also guide students to discover the advantages of the nursing profession to increase their personal willingness, positive emotions, and initiative. This will lead to an enhancement of core competencies. Meanwhile, educators should identify the motivations behind students’ choices. Those influenced by family pressure need help internalizing the value of their careers. For those who choose nursing voluntarily, educators should reinforce their motivation and enhance their sense of professional identity to promote the development of their core abilities. Nursing intern students whose intended employment was “other job” scored significantly lower on core competencies. Conversely, students who viewed nursing as their future career scored higher on clinical competencies [61]. Those students who are willing to remain in nursing‐related professions after their final clinical placement have clearer goals and plans. They will focus on developing their core competencies during the placement to meet the position’s basic requirements. Nursing intern students are the nursing staff of the future. Therefore, clinical administrators should take steps to establish a favorable climate for clinical teaching [6] and help nursing intern students have positive experiences to attract and retain nursing staff for healthcare organizations [63].

### 4.3. The Relationship Between Core Competencies of Nursing Intern Students and Spiritual Health

Correlation analysis and hierarchical multiple regression analysis showed that spiritual health was significantly positively correlated with core competencies. This discovery can be explained by the competency iceberg model theory. The iceberg model of competence provides a hierarchical perspective to illustrate the complex relationship between the spiritual health and core competencies of nursing intern students. Just as the model assumes, ability traits are manifested in a two‐layer structure: explicit attributes (knowledge and skills) and implicit personal attributes (self‐concept, traits, and motivation), the latter of which drives the acquisition, application, and improvement of the former [40]. It is obvious that spiritual health, as a complex that can promote an individual’s life meaning, spiritual connection, and core value recognition [29], is a key implicit factor that exerts influence at both levels of the iceberg model to shape the core competencies of nursing intern students. Among the implicit attributes of the model, the significance of personal traits such as self‐worth, professional identity, and motivation for the development of core competencies is mainly emphasized [39]. Previous studies have confirmed that spiritual health plays a significant role in shaping the correct self‐perception of nursing intern students and promoting the formation of core professional values [64, 65]. Through spiritual health, nursing intern students can better find the meaning and purpose of life and work, align their actions with personal values, and gain a higher sense of professional identity in professional practice, acting for the real interests of patients. A study by Yao et al. [19] found that nursing intern students with higher self‐efficacy and professional identity could participate in professional activities more consciously and demonstrate stronger self‐study abilities. In the iceberg model, what is reflected is the influence of spiritual health on the personal trait of “core values,” thereby driving intrinsic motivation to promote the development of core capabilities, rather than merely relying on external incentives such as material rewards and recognition from others [66]. This suggests that spiritual health can lay an internal driving foundation for the development of core competencies. In addition, spiritual health advocates that individuals should establish connections and a sense of belonging with others [67]. In the clinical setting, nursing intern students with a high level of spiritual health can usually establish harmonious relationships with patients, colleagues, and teachers more easily [68, 69]. Previous studies have shown that such positive interpersonal relationships can help nursing intern students obtain more learning resources and opportunities, promoting their continuous accumulation of medical knowledge and nursing experience, as well as learning clinical operation and communication skills [70]. This is an important process for cultivating core competencies. In the iceberg model of competence, what is reflected is the influence of spiritual health on the explicit attributes of ability (knowledge and skills) through the process of connecting with others. In addition, studies have shown [71, 72] that nursing intern students with good spiritual health conditions, through profound reflection on the meaning of life, often possess higher empathy and empathetic communication skills, can establish positive interpersonal relationships with patients, and are better at understanding and responding to patients’ care and spiritual needs. This is due to the humanistic attribute of spiritual health, which endows nursing intern students with a higher level of spiritual health with a higher ability to care and provide spiritual care [41, 73]. These are also the core competencies that nursing intern students should possess. Therefore, schools and hospitals should design targeted intervention measures, such as life meaning reflection and spiritual care training courses, to improve the spiritual health of nursing intern students in several aspects, including cultivating good interpersonal relationships, clarifying the meaning of life and career goals, and helping nursing intern students correctly understand themselves [74], so as to strengthen the implicit foundation of their abilities and promote the development of their core competencies.

Finally, this study found that the religious belief dimension of spiritual well‐being among nursing intern students scored the lowest, consistent with previous research [74, 75], and showed a weak correlation with core competencies. This may be due to the limitations of the cultural adaptation of the research instrument: The descriptions of religious belief in the scale are mainly based on Western cultural backgrounds. However, Chinese nursing intern students generally have relatively limited contact with or understanding of Western religious activities and beliefs. This is closely related to China’s longstanding atheistic education and the dialectical materialist worldview [75]. Therefore, to more accurately assess the spiritual well‐being of Chinese nursing intern students, it is recommended that researchers fully consider culturally appropriate adjustments in the process of scale sinicization or the development of localized scales. In the religious belief dimension, Chinese traditional cultural and spiritual elements, such as Confucianism, Taoist philosophy, and Buddhism, should be integrated to create a spiritual assessment tool that aligns more closely with the local cultural context [76].

### 4.4. Limitations

This study has the following limitations: First, the study was conducted in five tertiary hospitals in China. Therefore, the results may not be generalized to nursing intern students in other countries. At the same time, due to restrictions imposed by the policies aimed at preventing and controlling the spread of the COVID‐19, cross‐regional sampling faced practical difficulties. Sampling was only conducted in Sichuan Province, which limited the regional nature of the sample and failed to reflect the situation of nursing intern students in hospitals across China. As a result, the research findings could not be generalized to a broader context. Therefore, future research will adopt multicenter and stratified sampling and try to include medical institutions from all over the country to improve the generalizability of the results. Second, this study is a cross‐sectional study and lacks long‐term follow‐up data. Therefore, it is temporarily impossible to establish a causal relationship and an influencing mechanism between spiritual health and core competencies. It can only confirm their association. In the future, the impact of spiritual health can be further verified through cohort studies with larger sample sizes or experimental designs that control confounding variables, in order to make up for the deficiencies of the current research. Third, only 42.9% of the variance in spiritual health was explained. Future research could explore other predictors and incorporate more indicators and variables related to core competencies in the research design, such as prior academic performance, mentorship quality, or clinical workload, to better explain the level of core competencies and the influencing factors of nursing intern students.

## 5. Conclusions

This study mainly confirms that the core competencies of nursing intern students in China are at a medium level, among which critical thinking and reasoning abilities are the weakest components. This discovery suggests that nursing managers and educators should pay attention to the systematic cultivation of the core competencies of nursing intern students. Meanwhile, through methods such as scenario simulation teaching, reflective practical training, and clinical experience inheritance, students’ ability to transform knowledge into clinical practice is strengthened, thereby enhancing their critical thinking and reasoning skills, achieving a comprehensive improvement in their overall abilities, and providing high‐quality talents for the nursing industry. Furthermore, this study found that spiritual health has a positive influence on improving the core competencies of nursing intern students, which provides an intervention perspective for the improvement of core competencies. Schools and hospitals should incorporate intervention methods such as reflection on the meaning of life and spiritual care training into their daily management to enhance the spiritual health level of nursing intern students and thereby strengthen their core competencies.

### 5.1. Implications for Nursing Management

Improved core competencies of nursing intern students translate into improved quality of education and quality of care. Therefore, nursing managers and educators should help nursing intern students manage and improve their spiritual health to better enhance their core competencies.

## Author Contributions

Limei Zhang: conceptualization. Limei Zhang, Xiaoying Zhong, and Jing Liu: methodology. Limei Zhang, Xiaoying Zhong, Jing Liu, and Tingting Yuan: formal analysis, writing–original draft preparation, and revision. Limei Zhang, Xiaoying Zhong, Jing Liu, Tingting Yuan, Wenli Yang, Meng Wang, and Ping Yuan: investigation. Limei Zhang, Jing Liu, Wenli Yang, Meng Wang, and Ping Yuan: data curation. Limei Zhang, Xiaoying Zhong, Jing Liu, Tingting Yuan, and Xing Ding: writing–review and editing. Limei Zhang and Xing Ding: supervision. Limei Zhang, Xiaoying Zhong, Jing Liu, and Tingting Yuan are the co‐first authors.

## Funding

This work was supported by grants from the Chengdu Medical College Key Project Undergraduate Education and Teaching Reform Research Project (Grant No. JG2024005) the Open Fund of Sichuan Provincial Key Laboratory of Philosophy and Social Sciences for Intelligent Medical Care and Elderly Health Management (Grant No. 24LHZLZS1‐03) and 2025 Undergraduate Education and Teaching Reform Project of Chengdu Medical College (Grant No. JG2025020).

## Disclosure

All authors approved the final version for submission.

## Conflicts of Interest

The authors declare no conflicts of interest.

## Data Availability

The data that support the findings of this study are available from the corresponding author upon reasonable request.
